# Predictive factors for therapeutic response to radioactive iodine therapy in pulmonary metastases from differentiated thyroid carcinoma

**DOI:** 10.1007/s11604-026-01982-y

**Published:** 2026-06-11

**Authors:** Ken Watanabe, Takao Igarashi, Yuki Hayakawa, Kimiyuki Uchihara, Takashi Kazama, Hiroya Ojiri

**Affiliations:** 1 Department of Radiology, The Jikei University School of Medicine, 3-25-8, Nishi-Shinbashi, Minato-ku, Tokyo 105-8461 Japan; 2 Department of Radiology, Tokyo Dental College Ichikawa General Hospital, 5-11-13 Sugano, Ichikawa, Chiba 272-8513 Japan; 3 Department of Breast/Thyroid/Endocrine Surgery, The Jikei University School of Medicine, 3-25-8, Nishi-Shinbashi, Minato-ku, Tokyo 105-8461 Japan

**Keywords:** Differentiated thyroid carcinoma, Pulmonary metastasis, Radioactive iodine therapy, Prognostic factors, Treatment response, Iodine uptake

## Abstract

**Purpose:**

To evaluate the therapeutic outcomes of radioactive iodine (RAI) therapy for pulmonary metastases from differentiated thyroid carcinoma (DTC) and identify clinical factors predictive of treatment response.

**Methods:**

This retrospective study included 186 patients with pulmonary metastases of DTC who underwent RAI at our institution between 2010 and 2024. The treatment response was assessed based on integrated radiological and biochemical criteria using CT, I-131 scintigraphy, and serum thyroglobulin levels. Patients were classified as disease control complete response (CR), partial response (PR), stable disease (SD), or progressive disease (PD). Clinical variables including age, sex, histological subtype, timing of pulmonary metastasis diagnosis, maximum lesion diameter, pulmonary RAI uptake score, initial administered activity, and history of adjuvant RAI therapy were evaluated. Univariate and multivariate logistic regression analyses were performed to identify independent predictors of treatment response.

**Results:**

The overall disease control rate (CR + PR + SD) was 23.1%. Younger age (< 55 years) and synchronous detection of pulmonary metastases at the time of thyroid surgery were identified as independent predictors of a favorable treatment response (odds ratio = 3.77 and 2.65, respectively). A strong positive correlation was observed between the pulmonary RAI uptake score and the therapeutic outcome (Spearman’s ρ = 0.683, *p* < 0.001). In contrast, the initially administered activity (100 vs. ≥ 150 mCi) was not significantly associated with treatment efficacy.

**Conclusion:**

Younger age and synchronous detection of pulmonary metastases were identified as independent predictors, whereas pulmonary RAI uptake was strongly correlated with favorable RAI responsiveness in patients with DTC. The identified predictors of disease control may assist in optimizing patient selection and counseling for RAI therapy.

## Introduction

Differentiated thyroid carcinoma (DTC) accounts for most thyroid malignancies, with papillary thyroid carcinoma (PTC) being the most common histological subtype. The global incidence of thyroid cancer has increased in recent decades, largely due to the widespread use of high-resolution imaging and the detection of small, asymptomatic tumors [1, 2]. Although DTC typically follows an indolent course and is associated with a favorable prognosis [3], it can metastasize via the lymphatic and hematogenous routes, with the lungs representing one of the most common sites of distant metastasis [4–6]. Given their frequency and biological heterogeneity, pulmonary metastases are clinically important for evaluating the therapeutic efficacy of radioactive iodine (RAI).

RAI therapy following total thyroidectomy and thyroid-stimulating hormone (TSH) suppression remains the cornerstone of DTC management, especially in patients with residual disease, locoregional recurrence, or distant metastases, including pulmonary involvement [7, 8]. Despite its established role, the therapeutic efficacy of RAI in pulmonary metastases varies, with a substantial subset of patients exhibiting suboptimal or absent responses. This variability represents a key clinical challenge and highlights the importance of identifying reliable determinants of the treatment response.

Recent improvements in diagnostic imaging have led to early detection of metastatic disease, and an increasing number of older patients are being referred for RAI therapy [9]. Furthermore, the variable iodine avidity of pulmonary metastases and constraints in specialized nuclear medicine resources underscore the need to refine the patient selection criteria. Younger patients and those with small micronodular metastases are known to derive greater benefits from RAI [6, 10–13]. Other potentially important prognostic factors, such as comorbidities, detailed lesional characteristics, optimal RAI dosing strategies, timing of metastasis detection, and quantitative assessment of radioiodine uptake have been limited in prior investigations because of methodological challenges and insufficient standardization. Moreover, many previous studies have been constrained by small sample sizes or heterogeneous patient populations, limiting their statistical power and impeding the robust identification of independent predictors. Therefore, the important factors that determine the therapeutic responsiveness remain insufficiently understood, and further investigation is required to optimize individualized treatment strategies for this patient population.

Addressing these knowledge gaps is essential for improving patient-specific therapeutic decision-making and optimizing the use of nuclear medicine resources. Therefore, this retrospective study aimed to evaluate disease control, defined as complete response (CR), partial response (PR), or stable disease (SD), following RAI therapy for pulmonary metastases from DTC at our institution, and to identify clinical and radiological factors associated with therapeutic efficacy, including those that have been underexplored or inadequately assessed in previous cohorts. By analyzing our institutional experience, this study aimed to clarify the role of patient age, timing of metastasis detection, administered RAI activity, and pulmonary RAI uptake scores as predictors of treatment response, thereby contributing to the evidence for optimizing RAI therapy and supporting more effective and resource-conscious management of patients with metastatic DTC.

## Materials and methods

### Patients

**Fig. 1:**
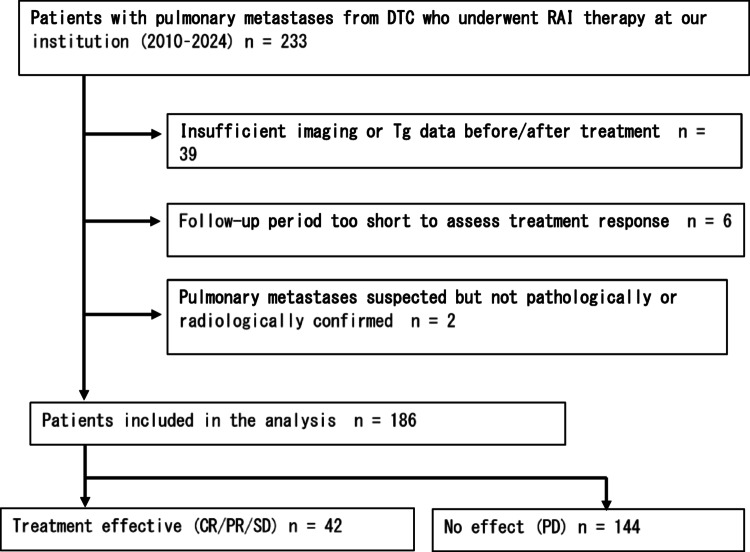
Flowchart of patient selection

RAI therapy was indicated for patients with confirmed pulmonary metastases based on computed tomography (CT) imaging, post-therapeutic I-131 scintigraphy from prior adjuvant therapy, or elevated TSH-stimulated thyroglobulin levels, following ATA guidelines. We retrospectively identified 233 patients with DTC and pulmonary metastases who received RAI at our institution between January 2010 and December 2024. Patients were excluded if (1) imaging or laboratory data, including serum thyroglobulin (sTg) levels, before and after treatment were insufficient (*n* = 39); (2) the follow-up period after initial RAI therapy was shorter than 6 months, which was considered insufficient to assess treatment response (*n* = 6); or (3) pulmonary metastases were suspected but not pathologically or radiologically confirmed (*n* = 2). After applying these criteria, 186 patients were included in this study. The patient selection process is illustrated in Fig. 1.

Of the 186 patients, 26 (14%) had received adjuvant RAI therapy following total thyroidectomy. Adjuvant therapy was administered to high-risk patients based on the ATA risk stratification, typically to those with T4 disease, extensive lymph node involvement (N1b), extrathyroidal extension, or aggressive histological features. The administered dose for adjuvant therapy was evenly distributed: 30 mCi in 13 patients (50%) and 100 mCi in 13 patients (50%), as determined according to the institutional protocols. The median interval from adjuvant therapy to the diagnosis of pulmonary metastases was 85.5 months (range: 12–240 months). Treatment decisions for pulmonary metastases were uniformly based on structural disease evidence on CT imaging, elevated thyroglobulin levels under TSH stimulation, and overall clinical assessment. Treatment strategies were not systematically stratified according to prior adjuvant therapy outcomes or post-adjuvant scintigraphy findings.

### RAI therapy protocol

Pre-therapeutic diagnostic scans were not routinely performed to avoid potential “stunning” effects and to ensure treatment decisions were not restricted based on predicted radioiodine avidity, resulting in an unselected consecutive cohort. All patients received RAI therapy after thyroid hormone withdrawal to increase the endogenous TSH levels. Levothyroxine (100–200 µg/day) was discontinued 4 weeks before therapy and replaced with triiodothyronine (10–20 µg/day) for 2 weeks to facilitate TSH elevation. Patients adhered to a strict low-iodine diet for two weeks before RAI therapy. A TSH level ≥ 30 mU/L was considered adequate for treatment initiation.

The administered activity of I-131 was determined according to the institutional policy and clinical period. Initially, the maximum allowable activity per treatment was limited to 3700 MBq (100 mCi). In recent years, the upper limit has increased to 7400 MBq (200 mCi) per treatment. Accordingly, the patients were retrospectively classified into two groups based on the initially administered activity: 100 mCi and ≥ 150 mCi.

None of the patients received recombinant human thyrotropin alpha (TSH) as preparation for RAI therapy. Additional RAI treatments were administered as clinically indicated based on disease status and physician judgment.

### Evaluation of treatment response

Treatment response was assessed 6–12 months after the initial RAI therapy, as RAI therapeutic effects develop gradually, and this timeframe represents the optimal period for capturing tumor regression and biochemical changes according to ATA guidelines [8].

The treatment response was assessed using CT and I-131 whole-body scintigraphy, and sTg levels were measured under TSH stimulation. For patients with positive anti-Tg antibody results, the treatment response was evaluated based solely on imaging findings. The therapeutic effect was comprehensively evaluated by combining serum thyroglobulin levels and RAI uptake according to the ATA guidelines, and the following four categories were defined [8].

Complete response (CR): complete disappearance of pulmonary lesions on imaging and Tg levels below the detection limit.

Partial response (PR): Apparent reduction in the size of pulmonary lesions or decreased RAI uptake, accompanied by a ≥ 50% decrease in Tg levels.

Stable disease (SD): No significant change in pulmonary lesion size (no change to a slight decrease in RAI uptake), accompanied by a < 50% decrease in Tg level.

Progressive disease (PD): appearance of new pulmonary lesions, enlargement of existing lesions, increased RAI uptake, or a ≥ 50% increase in Tg levels. In cases of mixed response, in which some lesions regressed while others progressed, patients were classified as having PD if any new lesions appeared, existing lesions showed clear enlargement, or thyroglobulin levels increased by ≥ 50%, regardless of the response of other lesions.

For statistical analyses, CR, PR, and SD were collectively defined as “response,” whereas PD was categorized as “no response.” As the study population consisted of patients with pulmonary metastases requiring RAI therapy in whom disease progression would be expected without effective treatment, stable disease was considered a clinically meaningful therapeutic outcome reflecting disease control.

### Predictive factors

The following clinical and imaging factors were evaluated as potential predictors of treatment response: age, sex, histological subtype (papillary carcinoma vs. follicular carcinoma), timing of diagnosis of pulmonary metastasis (synchronous with primary surgery vs. metachronous), maximum diameter of the pulmonary lesions, degree of pulmonary uptake on post-therapeutic RAI scintigraphy, initial RAI activity (mCi), and history of adjuvant RAI therapy.

**Fig. 2:**
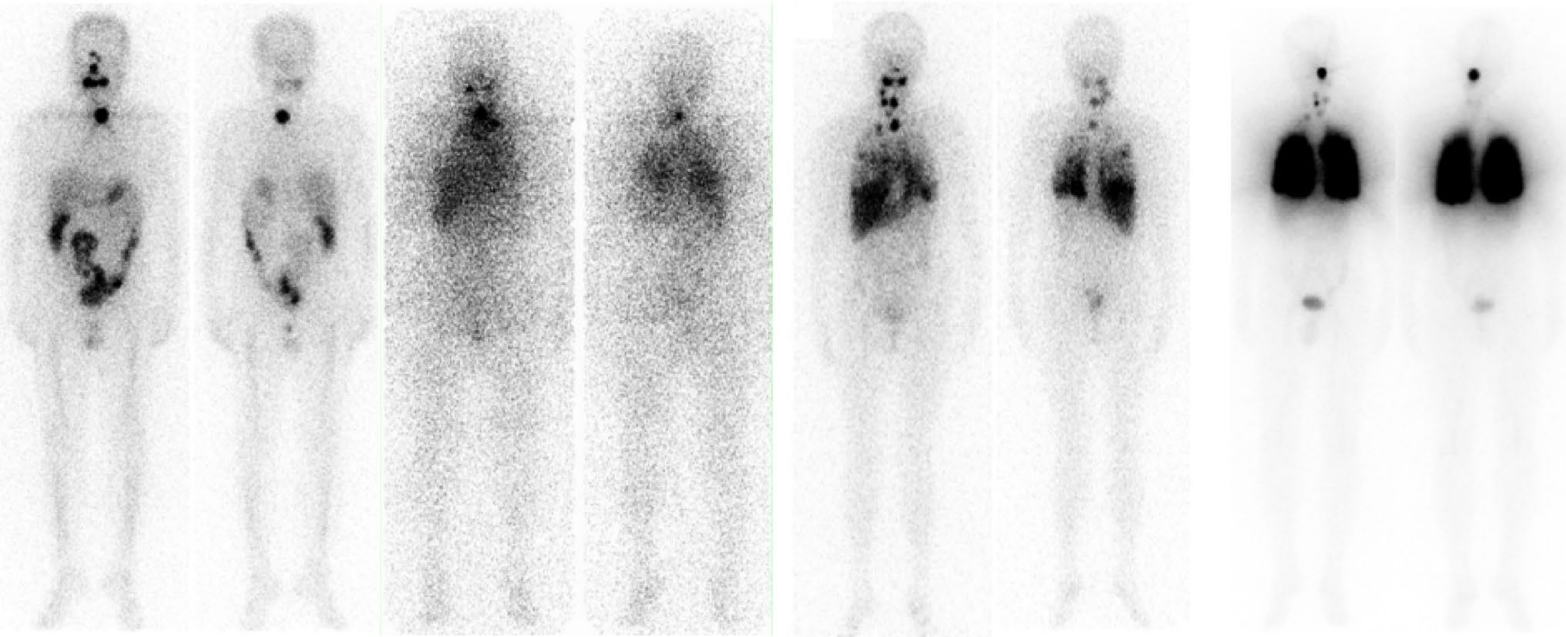
Representative examples of pulmonary radioactive iodine (RAI) uptake scores. The degree of pulmonary RAI uptake was visually classified into four categories by comparing the thyroid bed uptake: a score of 0 (far left), no pulmonary uptake; score 1 (center-left): faint uptake lower than the thyroid bed; score 2 (center-right): moderate uptake similar to the thyroid bed; score 3 (far right): intense uptake higher than the thyroid bed

The degree of pulmonary RAI uptake was classified into four categories by comparison with thyroid bed uptake: score 0, no uptake; score 1, mild uptake; score 2, moderate uptake; and score 3, intense uptake (Fig. 2). All images were independently reviewed by two board-certified nuclear medicine physicians and the final classification was determined by consensus.

### Imaging protocol

Post-therapeutic I-131 whole-body scintigraphy was performed 5–7 days after RAI administration using a Discovery NM/CT 630 system (GE Healthcare, Chicago, IL, USA). A high-energy general-purpose collimator was used in this study. The acquisition parameters included a 364-keV photopeak with a ± 7.5% energy window, a matrix size of 256 × 256, a pixel size of 2.2 mm, and a scan speed of 6–10 cm/min.

### Statistical analysis

Patients were divided into two groups according to their treatment outcomes: a disease control group (CR, PR, and SD) and a progressive disease (PD) group. Categorical variables were compared using the chi-square test or Fisher’s exact test, as appropriate. Continuous variables were analyzed using the Mann–Whitney U test. Variables showing statistical significance in the univariate analyses and clinically relevant factors were included in the multivariate logistic regression model to identify independent predictors of disease control. However, to ensure model stability and statistical validity, variables that demonstrated quasi-complete separation (causing non-convergence of the maximum likelihood estimation) or those that violated the Events Per Variable rule (requiring 10–15 outcome events per predictor) were excluded from the final model. The final multivariate analysis included age, sex, timing of metastasis detection, history of adjuvant RAI therapy, pulmonary lesion size, and initially administered activity. The odds ratios (ORs) and 95% confidence intervals (CIs) for each variable were calculated. Statistical significance was set at *p* < 0.05. All statistical analyses were performed using the SPSS Statistics software (version 25; IBM Corp., Armonk, NY, USA).

### Ethical considerations

This retrospective study was conducted in accordance with the principles of the Declaration of Helsinki. The study protocol was reviewed and approved by the Institutional Review Board (IRB) of our hospital (approval no. 37–091 [12728]). The requirement for written informed consent was waived due to the retrospective nature of the study.

## Results

### Overall treatment response

Among the 186 patients included in the analysis, 6 (3.2%) achieved CR, 31 (16.7%) achieved PR, 5 (2.7%) were classified as having SD, and 144 (77.4%) as having PD. The overall disease control rate (CR + PR + SD) was 23.1%.

### Univariate analysis

The baseline clinical and pathological characteristics according to treatment response are summarized in Table 1. Responders tended to be younger than non-responders (median age: 56 vs. 67 years; *p* = 0.07) with borderline statistical significance. The histological subtypes differed significantly between the groups, with follicular carcinoma and poorly differentiated carcinoma being more common among responders and non-responders, respectively (*p* = 0.001).

**Table 1:** Clinical characteristics of patients with and without treatment response (*n* = 186)

Variables	No response (*n* = 144)	Response (*n* = 42)	*P* value
Age (years)	20–87 (median 67, mean 62.9)	16–78 (median 56, mean 56.0)	0.07
Sex (male/female)	50/94	11/31	0.353
Histopathology			0.001
Papillary	128	28	
Follicular	12	13	
Poorly	4	1	
Interval to diagnosis (months, range, median)	0–348, 55.6	0–360, 37.8	0.004
T stage			0.063
T1	9	4	
T2	13	4	
T3	45	17	
T4	33	2	
N stage			0.067
N0	10	7	
N1a	24	8	
N1b	68	13	
History of adjuvant RAI therapy (no/yes)	120/24	40/2	0.074
Iodine excretion (range/mean/median)	10–3874/163.7/56.5	24–182/64.3/57.0	0.51
Size of pulmonary metastases			0.54
< 5 mm	71	21	
5–10 mm	48	11	
≥ 10 mm	25	10	
Degree of pulmonary uptake			< 0.001
No uptake (score 0)	93	0	
Mild faint (score 1)	33	6	
Moderate uptake (score 2)	17	20	
Intense uptake (score 3)	1	16	
Bone metastasis (no/yes)	134/10	32/10	0.004
Administered activity			0.487
≤100 mCi	120	37	
≥150 mCi	24	5	

Response: CR, PR, or SD; Non-response: PD

The synchronous detection of pulmonary metastases at the time of thyroid surgery was significantly associated with a favorable treatment response (median interval, 0 vs. 33 months; *p* = 0.004). No significant differences were observed in sex distribution (*p* = 0.353), T stage (*p* = 0.063), N stage (*p* = 0.060), urinary iodine excretion (*p* = 0.510), or maximum pulmonary lesion size (*p* = 0.540) between the two groups.

**Fig. 3:**
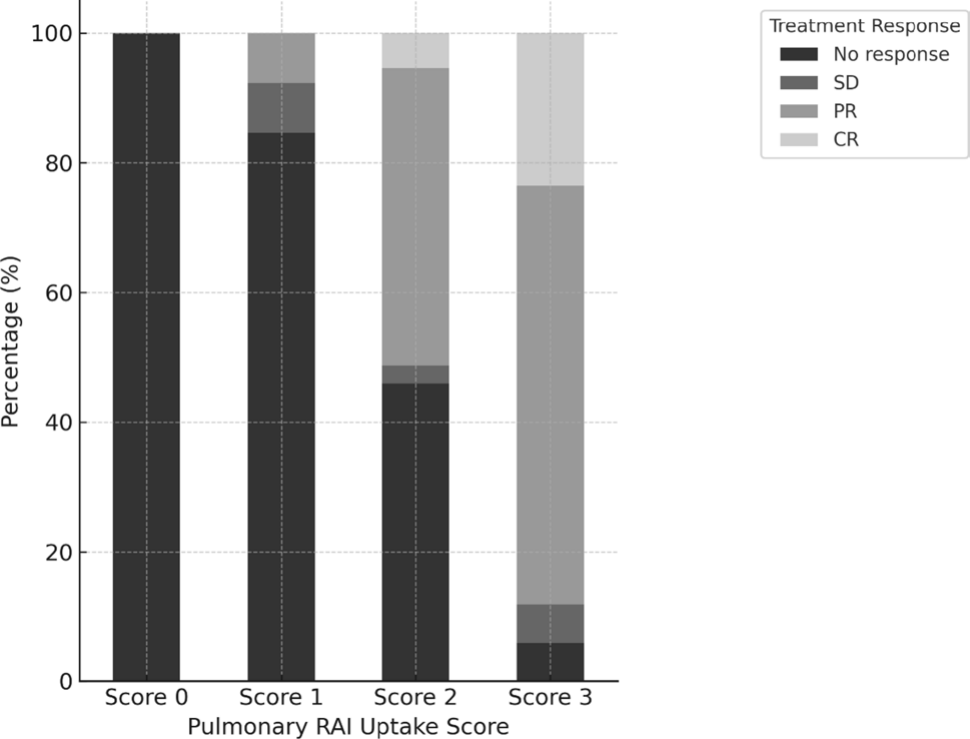
Distribution of treatment response by pulmonary RAI uptake score (percentage). The proportions of treatment responses (complete response [CR], partial response [PR], stable disease [SD], and progressive disease [PD]) were determined based on the pulmonary RAI uptake score (0–3). Higher RAI uptake scores were associated with increased CR and PR frequencies. A significant positive correlation was observed between the RAI uptake score and treatment response (Spearman’s rank correlation coefficient, ρ = 0.683; *p* < 0.001)

The degree of pulmonary RAI uptake was strongly associated with the treatment response (*p* < 0.001). Patients with higher uptake scores (2–3) were more frequently classified as responders, whereas those with no uptake or faint uptake (scores 0–1) were predominantly classified as non-responders (Fig. 2). A significant positive correlation was observed between the pulmonary RAI uptake score and treatment response (Spearman’s rank correlation coefficient ρ = 0.683, *p* < 0.001, Fig. 3).

Bone metastases were significantly more frequent in the non-responder group (*p* = 0.004). A history of adjuvant RAI therapy tended to be less frequent among the responders, although the difference was not statistically significant (*p* = 0.074). The initially administered activity (100 vs. ≥150 mCi) was not significantly associated with the treatment response (*p* = 0.487).

### Multivariate analysis

Owing to statistical constraints, including quasi-complete separation for pulmonary RAI uptake (no responders among 93 patients with a score of 0) and limited outcome events (*n* = 42), multivariate logistic regression analysis was restricted to variables with stable parameter estimation. The results of the multivariate logistic regression analysis are shown in Table 2.

**Table 2:** Multivariate logistic regression analysis of predictive factors for RAI treatment response

Independent variables	OR	95% CI (Lower)	95% CI (Upper)	*P* value
Age < 55 years	3.77	1.63	8.69	0.002
Sex	1.58	0.67	3.71	0.299
Simultaneous diagnosis of pulmonary metastasis with primary surgery	2.65	1.04	6.75	0.042
No history of adjuvant RAI therapy	2.8	0.58	13.49	0.199
Pulmonary metastatic lesions < 5 mm	0.6	0.22	1.62	0.314
Initial administered activity ≥ 150 mCi	1.13	0.36	3.53	0.84

OR = odds ratio; CI = confidence interval; RAI = radioactive iodine

Age < 55 years was identified as an independent predictor of a favorable response to RAI therapy (OR 3.769; 95% CI 1.634–8.694; *p* = 0.002). Moreover, synchronous diagnosis of pulmonary metastases at the time of thyroid surgery was independently associated with treatment response (OR 2.646; 95% CI 1.037–6.752; *p* = 0.042).

Other factors, including sex (OR 1.575; 95% CI 0.669–3.709; *p* = 0.299), history of adjuvant RAI therapy (OR 2.800; 95% CI 0.581–13.491; *p* = 0.199), pulmonary lesion size < 5 mm (OR 0.600; 95% CI 0.222–1.621; *p* = 0.314), and initial administered activity ≥ 150 mCi (OR 1.125; 95% CI 0.358–3.532; *p* = 0.840), were not significantly associated with the treatment response.

### Sensitivity analysis

Sensitivity analyses were performed to assess the robustness of the findings against potential confounding factors.

To assess the potential confounding effects of prior adjuvant RAI therapy on uptake scoring, we performed a subgroup analysis of patients without a history of adjuvant therapy (*n* = 160). The strong positive correlation between pulmonary RAI uptake score and treatment response was virtually identical to the overall cohort (Spearman’s ρ = 0.684, *p* < 0.001), confirming that our primary findings were not influenced by potential scoring bias related to reduced thyroid bed activity.

Second, to address the potential confounding effects of bone metastases on the thyroglobulin-based treatment response assessment, we performed a subgroup analysis limited to patients with isolated pulmonary metastases (*n* = 166). The overall disease control rate was 19.3% (32/166). The correlation between pulmonary RAI uptake score and treatment response remained highly significant (Spearman’s ρ = 0.664, *p* < 0.001). In multivariate logistic regression analysis, younger age (< 55 years) demonstrated enhanced predictive value (OR = 6.33, 95% CI 2.37–16.95, *p* < 0.001), whereas synchronous detection maintained a consistent effect size (OR = 2.78, 95% CI 0.94–8.22, *p* = 0.065). The borderline significance of synchronous detection reflects the reduced statistical power with a smaller sample size, whereas the maintained effect size confirms its clinical relevance. These findings confirm the robustness and consistency of our primary conclusions across different patient subsets.

## Discussion

This retrospective study evaluated the outcomes of RAI therapy in 186 patients with pulmonary metastases from DTC and investigated the potential predictors of treatment response. The overall disease control rate (complete as CR, PR, or SD) was 23.1%. Multivariate logistic regression analysis identified two independent predictors of favorable treatment outcomes: age < 55 years and synchronous diagnosis of pulmonary metastases at the time of thyroid surgery. Furthermore, these results demonstrated a strong correlation between pulmonary RAI uptake and treatment response, whereas the initially administered activity (100 vs. ≥150 mCi) was not significantly associated with therapeutic efficacy.

An overall disease control rate of 23.1% reflects our unselected patient population. As pre-therapeutic diagnostic scanning was not performed, our cohort naturally included substantial non-avid diseases, with 50% (93/186) demonstrating no therapeutic uptake (score 0). Among patients with demonstrable uptake (scores 1–3), the disease control rate was 45.2% (42/93), which is consistent with previous reports [8]. This approach reflects the real-world clinical practice and makes our findings applicable to routine patient care. The robustness of our findings was confirmed through comprehensive sensitivity analysis. When restricted to patients without bone metastases (*n* = 166), the correlation between pulmonary RAI uptake and treatment response was maintained (ρ = 0.664, *p* < 0.001). Notably, the predictive value of younger age was enhanced in this subgroup (OR = 6.33 vs. 3.77 in the overall cohort), suggesting that removal of bone metastases as a confounding factor strengthens the clarity of age-related prognostic differences. Synchronous detection maintained a consistent effect size (OR = 2.78 vs. 2.65) with borderline statistical significance (*p* = 0.065) attributable to a reduced sample size rather than a loss of clinical relevance. The consistency of effect sizes and directions across both the overall cohort and subgroup analyses strongly supports the validity and generalizability of our identified predictive factors.

These results are consistent with those of previous studies, indicating that younger patients are more likely to benefit from RAI therapy and reaffirming the importance of age as a prognostic factor in DTC [7, 9, 11, 14, 15]. The degree of pulmonary uptake is also recognized as a critical determinant of RAI responsiveness. These results further support this association by quantitatively confirming a strong positive correlation [16]. The strong association between pulmonary RAI uptake and responsiveness likely reflects underlying differences in tumor differentiation and sodium/iodide symporter (NIS) expression. Well-differentiated metastatic lesions tend to retain higher NIS expression, resulting in greater iodine avidity and better therapeutic outcomes, whereas advanced or dedifferentiated lesions characteristically downregulate NIS or mislocalize it away from the plasma membrane, leading to the loss of iodine avidity and RAI refractoriness [17]. This framework is biologically plausible and consistent with reports linking MAPK pathway activation to reduced NIS function and with the clinical efficacy of redifferentiation strategies aimed at restoring iodine uptake [14, 18–21]. Therefore, quantitative assessment of pulmonary RAI uptake may serve as a functional surrogate marker of tumor differentiation and RAI responsiveness, providing prognostic information beyond conventional clinical parameters.

In contrast to these biologically driven factors, the impact of the administered RAI activity represents a therapeutic strategy–related issue rather than an intrinsic tumor characteristic. Regarding the administered activity, there has been considerable debate over whether higher doses improve outcomes in patients with metastatic disease. In our cohort, no significant difference in disease control was observed between patients initially treated with 100 mCi and those treated with ≥ 150 mCi. Importantly, this analysis focused only on initially administered activity and did not account for cumulative doses. Cumulative activity was deliberately avoided because patients who respond well may require fewer treatments, and thus receive a lower total dose, whereas those with suboptimal responses tend to undergo repeated administration, resulting in higher cumulative activity. Therefore, cumulative dose may not necessarily be a reliable surrogate for treatment efficacy. Nonetheless, it is possible that higher initial doses could reduce the total number of treatment sessions required to achieve disease control, which could be clinically meaningful by decreasing the treatment burden on patients. Further studies are required to address this issue.

This study found that synchronous detection of pulmonary metastases was associated with better outcomes than metachronous detection. Although this seems counterintuitive, metastases at diagnosis may indicate a more aggressive disease, which may be explained by several factors. Patients with synchronous metastases, especially those with large or symptomatic primary tumors, often undergo more thorough preoperative imaging, enabling earlier detection of lung lesions. Conversely, metachronous metastases typically arise in patients with initially smaller or less aggressive tumors, leading to the delayed identification of metastases owing to less extensive initial evaluations. Over time, these lesions may undergo clonal evolution and dedifferentiation, reducing iodine avidity and the therapeutic response. Thus, better outcomes in synchronous cases likely reflect a time-dependent dedifferentiation process, in which later-detected metastases respond less to RAI therapy. These findings, supported by recent studies [22], suggest that the timing of metastasis detection and the associated disease evolution play a more critical role in RAI responsiveness than initial tumor aggressiveness. Further research is required to clarify the relationship between disease biology and temporal changes in metastatic lesions.

Furthermore, although the histological subtype showed a significant association with disease control in univariate analysis, it was not included in the multivariate logistic regression model. This decision was based on the markedly uneven distribution of histological types in our cohort, particularly the small number of patients with follicular and poorly differentiated carcinomas, which may have introduced model instability or overfitting. Nonetheless, univariate findings suggest that tumor histology may influence RAI responsiveness, potentially reflecting intrinsic differences in tumor differentiation or expression of the sodium/iodide symporter. Larger studies with more balanced histological distributions are required to clarify the independent contributions of the histological subtypes to RAI treatment outcomes.

Although the history of adjuvant RAI therapy did not reach statistical significance in this study, the observed trend is noteworthy and provides important insights into disease biology. Patients with a history of adjuvant therapy showed markedly poorer response rates, with 92.3% (24/26) classified as nonresponders. Notably, only 4.8% (2/42) of the responders received prior adjuvant therapy, compared to 16.7% (24/144) of the non-responders. Patients who develop pulmonary metastases despite having previously undergone adjuvant therapy may harbor lesions that are intrinsically refractory to RAI. The prolonged median interval from adjuvant therapy to pulmonary metastasis diagnosis (85.5 months) suggests that such refractory diseases may represent gradual dedifferentiation or clonal evolution toward RAI refractoriness rather than primary treatment failure, thereby explaining the tendency toward poorer outcomes in this subgroup.

From a clinical perspective, integrating these predictive factors—patient age, timing of metastasis detection, and pulmonary RAI uptake—may enhance individualized treatment planning. In selected cases, patients predicted to have poor disease control or RAI responsiveness may be considered for early alternative strategies such as tyrosine kinase inhibitors or redifferentiation therapy [21]. Future prospective studies with molecular profiling may further elucidate the biological mechanisms underlying RAI resistance and help refine risk-adapted management strategies for metastatic DTC.

### Limitations

This study has several limitations. First, this was a retrospective analysis conducted at a single institution, which may have limited the generalizability of the findings. Second, the follow-up period varied among patients, and survival outcomes such as progression-free survival or overall survival were not systematically assessed. Third, the histological subgroups were unevenly distributed, with relatively few patients with poorly differentiated carcinomas, which may have influenced the statistical power to detect histology-related effects. Fourth, the analysis of the administered activity was restricted to the initial treatment dose without considering cumulative activity. Although this approach was chosen to minimize confounding by the treatment course, it limited our ability to fully evaluate the impact of RAI dosage on long-term outcomes. Fifth, the treatment response was evaluated based on imaging findings and changes in serum Tg levels, which may be affected by assay variability or the presence of anti-TgAb. Although anti-Tg antibody–positive patients were assessed using imaging alone, some degree of measurement bias cannot be ruled out. Sixth, the visual scoring of pulmonary RAI uptake, although performed by two experienced nuclear medicine physicians in consensus, remains a semi-quantitative assessment and may be subject to inter-observer variability. Finally, the potential selection bias and unmeasured confounding factors inherent to retrospective studies cannot be excluded. Moreover, the treatment response in this study does not necessarily equate to long-term clinical benefits, and future studies with standardized follow-up for survival outcomes are warranted.

## Conclusions

This retrospective study demonstrated that younger age and synchronous detection of pulmonary metastases at the time of thyroid surgery were independently associated with favorable disease control after RAI therapy in patients with DTC and pulmonary metastases. Additionally, pulmonary RAI uptake was strongly correlated with treatment response. In contrast, initially administered activity did not significantly correlate with disease control. The identified predictors of disease control may assist in refining patient selection and counseling in clinical settings. Nevertheless, prospective multicenter studies are warranted to validate these results and to clarify the role of RAI therapy in the clinical management of metastatic DTC.

## Data Availability

The datasets generated and/or analyzed during the current study are not publicly available because of patient privacy and ethical restrictions but are available from the corresponding author upon reasonable request.
